# Significance of the Tks4 scaffold protein in **bone tissue homeostasis**

**DOI:** 10.1038/s41598-019-42250-6

**Published:** 2019-04-08

**Authors:** Virag Vas, Tamás Kovács, Szandra Körmendi, Andrea Bródy, Gyöngyi Kudlik, Bálint Szeder, Diána Mező, Dóra Kállai, Kitti Koprivanacz, Balázs L. Merő, Metta Dülk, József Tóvári, Péter Vajdovich, Ş. Neslihan Şenel, Ilknur Özcan, Zsuzsanna Helyes, Csaba Dobó-Nagy, László Buday

**Affiliations:** 1 Institute of Enzymology, Research Centre for Natural Sciences, Hungarian Academy of Sciences, Budapest, Hungary; 2 Department of Prosthodontics, Faculty of Dentistry, Semmelweis University, Budapest, Hungary; 3 Department of Oral Diagnostics, Faculty of Dentistry, Semmelweis University, Budapest, Hungary; 4 Department of Experimental Pharmacology, National Institute of Oncology, Budapest, Hungary; 5 Department and Clinic of Internal Medicine, Szent Istvan University, Faculty of Veterinary Science, Budapest, Hungary; 6 Oral and Maxillofacial Radiology Department, Faculty of Dentistry at Istanbul University, Istanbul, Turkey; 7 University of Pécs, Medical School, Department of Pharmacology and Pharmacotherapy, János Szentágothai Research Centre, Molecular Pharmacology Research Team & Centre for Neuroscience, Pécs, Hungary; 8 Department of Medical Chemistry, Semmelweis University Medical School, Budapest, Hungary

## Abstract

The main driver of osteoporosis is an imbalance between bone resorption and formation. The pathogenesis of osteoporosis has also been connected to genetic alterations in key osteogenic factors and dysfunction of bone marrow mesenchymal stem/stromal cells (BM-MSCs). Tks4 (encoded by the *Sh3pxd2b* gene) is a scaffold protein involved in podosome organization. Homozygous mutational inactivation of *Sh3pxd2b* causes Frank-ter Haar syndrome (FTHS), a genetic disease that affects bone tissue as well as eye, ear, and heart functions. To date, the role of Tks4 in adult bone homeostasis has not been investigated. Therefore, the aim of this study was to analyze the facial and femoral bone phenotypes of *Sh3pxd2b* knock-out (KO) mice using micro-CT methods. In addition to the analysis of the *Sh3pxd2b*-KO mice, the bone microstructure of an FTHS patient was also examined. Macro-examination of skulls from Tks4-deficient mice revealed craniofacial malformations that were very similar to symptoms of the FTHS patient. The femurs of the *Sh3pxd2b*-KO mice had alterations in the trabecular system and showed signs of osteoporosis, and, similarly, the FTHS patient also showed increased trabecular separation/porosity. The expression levels of the *Runx2* and osteocalcin bone formation markers were reduced in the bone and bone marrow of the *Sh3pxd2b*-KO femurs, respectively. Our recent study demonstrated that *Sh3pxd2b*-KO BM-MSCs have a reduced ability to differentiate into osteoblast lineage cells; therefore, we concluded that the Tks4 scaffold protein is important for osteoblast formation, and that it likely plays a role in bone cell homeostasis.

## Introduction

Osteoporosis is a globally distributed, multifactorial metabolic bone disease characterized by dysregulation of bone tissue remodeling. Bone homeostasis is maintained via a fine balance between bone formation and bone resorption. Although the mechanisms of these osteoporotic processes have been extensively studied, the genetic factors^1,2^ and molecules that influence bone homeostasis are still under investigation^3–6^.

Scaffold proteins modulate cellular signaling by bringing regulatory proteins, enzymes or actin-organizing structures into close proximity^7^. The Tks protein family consists of large, multidomain scaffold proteins that are phosphorylated by the Src kinase, hence the acronym tyrosine kinase substrate (Tks)^8^. Tks4 contains four Src Homology 3 (SH3) domains, one Phox Homology domain (PXD) and several conserved linear motifs, e.g., proline-rich regions^9^. The Tks4-encoding gene was named *Sh3pxd2b* based on this domain structure. The primary function of the PX domain is to directly bind to membrane-associated phosphoinositides to tether Tks4 to the cell membrane. The SH3 domains mediate protein-protein interactions and are docking sites for other signaling components. Tks4 is involved in signal transduction in the EGFR pathway^10^. When Tks4 is phosphorylated by the EGFR-activated Src kinase, it interacts with cortactin to regulate the actin cytoskeleton. Tks4 is a key scaffold protein during podosome formation^11^; furthermore, it participates in reactive oxygen species (ROS) production by tumor cells^12^ and is necessary for mesenchymal stem/stromal cell (MSC) differentiation^13^.

Homozygous loss-of-function mutations in *Sh3pxd2b* gene lead to deletion or dysfunction of Tks4 protein, and lack of functional Tks4 causes a rare hereditary disease called Frank-ter Haar syndrome (FTHS, OMIM:249420)^14^. FTHS patients exhibit several distinct symptoms, including shortened and bowing long bones, kyphosis, cardiac deficiencies caused by valve or septal defects, craniofacial and dental abnormalities, and glaucoma^15–20^. Although some possible functions of Tks4 have been studied, the precise mechanisms through which Tks4 influences the FTHS-affected tissues remain elusive. To develop an animal model for studying FTHS, we generated a Tks4 knockout mouse strain with a targeted disruption of exons 5 and 6 in *Sh3pxd2b*^13^, and, as shown in our previous study, Tks4 was not detected in tissue from these *Sh3pxd2b*-KO mice^13^. Several phenotypes of the *Sh3pxd2b*-KO C57Bl/6 mice recapitulate FTHS pathologies, including craniomaxillofacial malformations, growth retardation (manifested by reduced long bone size and body mass), glaucoma and kyphosis^13^; furthermore, like FTHS patients, the Tks4-deficient mice develop heart failure. Although the abnormal bone morphology observed in the FTHS patients and the *Sh3pxd2b*-KO mice is a hallmark of the Tks4 mutant phenotype, the roles of Tks4 in *in situ* bone structure maintenance and bone homeostasis have not been investigated. To explore these open questions, we analyzed the bone structures of a unique adult FTHS patient and our Tks4-deficient mice in detail.

The analyses of the skulls and femurs of the *Sh3pxd2b*-KO mice and the mandibular bone of the FTHS patient revealed that the bone microstructure is disturbed in the absence of Tks4. Furthermore, the Tks4-mutant mice exhibited an osteoporotic phenotype, suggesting that the Tks4 scaffold protein is involved in osteoporotic processes.

## Results

### Cephalometric analysis of the craniofacial malformation in Tks4-deficient mice

**Figure 1:**
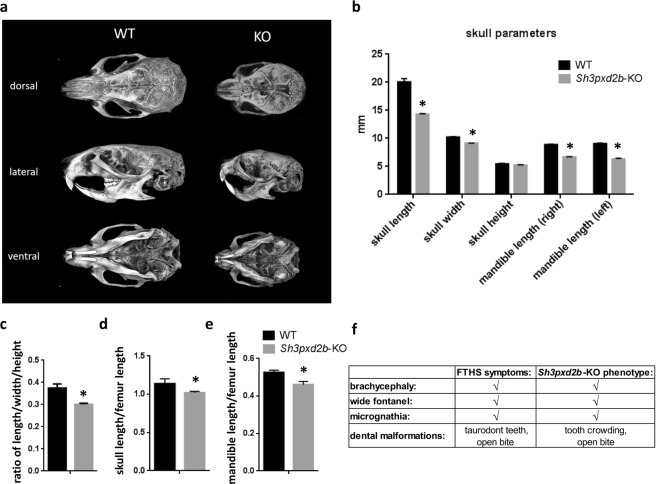
Cephalometric analysis of the cranial models of wild type and *Sh3pxd2b*-KO mice. (**a**) The dorsal, lateral and ventral views of representative 3D renderings of the skulls from 8-month-old wild type and *Sh3pxd2b*-KO mice show significant dysmorphology. (**b**) *Sh3pxd2b*-KO mice were compared to their genotypically wild type littermates to evaluate several parameters, including skull length, width, and height and mandible length. The anatomical landmark points used for the measurements are listed in the Methods section. Relative comparisons of the individual skull parameters: the skull length/width/height ratio (**c**), the skull length/femur length ratio (**d**) and the mandible length/femur length ratio (**e**). The differences in the data were analyzed by Student’s t-test and three *Sh3pxd2b*-KO and three wild type mice were used. ^*^p < 0.05. (**f**) Comparison of the dysmorphologies observed in skulls from *Sh3pxd2b*-KO mice with those reported in FTHS patients.

Micro-CT imaging was used to assess the craniofacial phenotypes of eight-month-old homozygous Tks4-deficient mice, and 3D reconstructions of the skulls were generated (Fig. 1a). The skulls of the *Sh3pxd2b*-KO mice exhibited significant reductions in length and width compared with the skulls of the wild type mice, and there was no difference in skull height. These observations indicate that loss of Tks4 leads to the formation of a domed cranium (Fig. 1b). Moreover, the skulls of the *Sh3pxd2b*-KO mice had a lower length:width:height ratio compared with that of the wild type mice, showing that the Tks4-deficient mice have a compressed skull shape (Fig. 1c). The most obvious difference between the wild type and *Sh3pxd2b*-KO skulls was a foreshortening of the anterior-posterior length. Comparison of the relative skull length to femur length ratio and the mandibular length to femur length ratio showed that the relative values in the *Sh3pxd2b*-KO mice were smaller than those in the wild type animals (Fig. 1d,e). Based on these observations, we concluded that the Tks4-deficient mice are, like FTHS patients, brachycephalic. Furthermore, the linear and relative mandibular lengths were also reduced in the *Sh3pxd2b*-KO mice, indicating micrognathia (Fig. 1a,c). We also analyzed 3D reconstructions of skulls from 28-day-old mice, and we observed similar ossification of the cranial base in the *Sh3pxd2b*-KO and wild type mice. The cranial and facial sutures were still visible and were not fused at the time of the examination in the mice in both groups. However, we observed wider anterior fontanelles in the young *Sh3pxd2b*-KO mice compared with those of the wild type mice. The *Sh3pxd2b*-KO mice also showed dental abnormalities, such as pronounced crowding and open bite in the molar region. However, we also noted that there was no marked malocclusion in *Sh3pxd2b*-KO mice in the sagittal dimension, suggesting that the reduction in midfacial and mandibular growth is roughly proportional. We performed systematic assessments of the cranioskeleton and searched for equivalent structures in the *Sh3pxd2b*-KO mice and the FTHS patient^17^. These analyses revealed several cranial malformations shared by the Tks4-deficient mice and the FTHS patient (Fig. 1f).

### Tks4 deficiency results in altered bone micro-morphology

**Figure 2:**
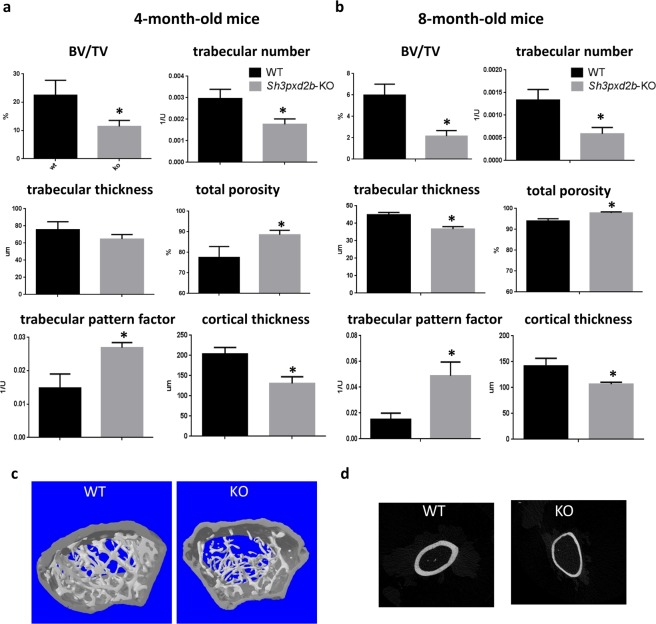
Bone architecture measurements of *Sh3pxd2b*-KO mice. (**a**) Analysis of the trabecular microarchitecture in 4-month-old wild type and *Sh3pxd2b*-KO mice based on Micro-CT data. The data are shown as the mean ± SEM of three mice in each group. (**b**) Micro-CT-based analysis of parameters describing the trabecular microarchitecture at a later time point (8 months old) measured in six wild type and six *Sh3pxd2b*-KO mice. Student’s t-test was used to assess the statistical significance of the difference between the means of the two groups. ^*^p < 0.05. (**c**) 3D images of the trabecular region of the femurs isolated from 8-month-old wild type and *Sh3pxd2b*-KO mice. (**d**) Representative 2D images of the cortical bone at the femoral mid-diaphysis from 8-month-old wild type and *Sh3pxd2b*-KO mice.

To determine the effects of loss of Tks4 on bone architecture, micro-CT imaging was performed on the femurs of 4- and 8-month-old *Sh3pxd2b*-KO mice (Fig. 2). The bone to tissue volume ratio (BV/TV) is a basic parameter for characterizing bone microstructure complexity. The imaging results showed that the BV/TV ratios were consistently higher in the metaphysis regions of the control femurs compared to those in the corresponding regions in the *Sh3pxd2b*-KO mice at the two time points analyzed (Fig. 2a,b). These results indicate a more than 50% reduction in bone structure per given tissue volume in the Tks4-deficient mice relative to the wild type mice, a phenotype that can indicate shape changes in the bone trabecular system (Fig. 2a,b). Consistent with this observation, the trabeculae in the bones from 8-month-old *Sh3pxd2b*-KO mice were significantly thinner and fewer in number (Fig. 2b,c) compared with those in the 8-month-old wild type mice. In the bones of the 4-month-old *Sh3pxd2b*-KO mice, the thickness of the trabeculae was not significantly reduced; however, pronounced thickening developed over time (Fig. 2a). This observation reflects a tendency for the *Sh3pxd2b*-KO bones to develop a more disconnected trabecular structure during aging. Furthermore, we found that the trabecular pattern factor was about 50% higher in the metaphyseal region of the Tks4-deficient animals compared with that of the wild type animals, indicating that the *Sh3pxd2b*-KO trabeculae did not form the intricate net-like pattern seen in the controls (Fig. 2a,b). Our data also revealed an increase in the total porosity in the metaphyseal region of *Sh3pxd2b*-KO mice compared with that of the wild type mice. The cortical thickness of the femoral bones was also reduced in the *Sh3pxd2b*-KO mice at every time point analyzed compared with the corresponding values in the wild type animals (Fig. 2a,b,d). Based on the results of the bone microstructure analysis, which showed that reduced trabecular number was accompanied by increased porosity, we concluded that Tks4 influences bone formation *in vivo* and that loss of this protein results in an osteoporotic bone phenotype.

**Figure 3:**
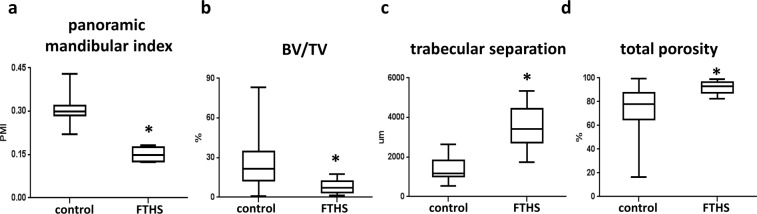
CBCT-derived parameters describing the bone architecture of the FTHS patient. (**a**) Panoramic mandibular index (PMI), (20 analyzed VOIs from 10 control individuals are included in the plot); (4 analyzed VOIs from one FTHS patient measured at two time points are included in the plot). (**b**) BV/TV (**c**) trabecular separation and (**d**) total porosity are presented using box-and-whisker plots (40 analyzed VOIs from 10 control individuals are included in the plot); (10 analyzed VOIs from one FTHS patient measured at two time points are included in the plot). Student’s t-test was used to determine the statistical significance of the differences between the means of the parameters. ^*^p < 0.05.

We next asked whether the bone microstructural changes described above could also be observed in an FTHS patient or if they were specific to the *Sh3pxd2b*-KO mice. To address this question, the maxillofacial region of a male FTHS patient was analyzed using available cone beam CT (CBCT) data. The CBCT data were obtained at the ages of 21 and 24 prior to surgical treatment to remove impacted teeth and alleviate dental complaints. Köse *et al*. previously described the dental malformations of this FTHS patient, which included micrognathia, hypoplasia of the teeth, taurodont teeth and an open bite^17^. In this study, we reanalyzed the CBCT data focusing not on the dental problems, but rather on the features of the mandibular bone and bone micro-morphology. We used radiomorphometric parameters, including the panoramic mandibular index (PMI), to identify osteoporotic changes, as a reduction in the volume of the mandibular cortex, which is reflected in the PMI, is a manifestation of low bone density. We found that the PMI was significantly reduced in the FTHS patient compared to that of the control individuals (Fig. 3a). Furthermore, the cancellous bone was also analyzed, and several structural differences were observed compared to the healthy control individuals. The BV/TV ratio was significantly lower in the FTHS patient compared with that of the control individuals, whereas the trabecular separation and the total porosity of the mandibular bone were higher in the FTHS patient (Fig. 3b–d).

Taken together, the altered appearance of the bone trabecular network in the *Sh3pxd2b*-KO mice and the abnormal mandibular bone structure of the FTHS patient suggest that Tks4 plays a role in bone remodeling and homeostasis.

### The potential involvement of osteoclast functions in the development of osteoporosis in *Sh3pxd2b-KO* mice

**Figure 4:**
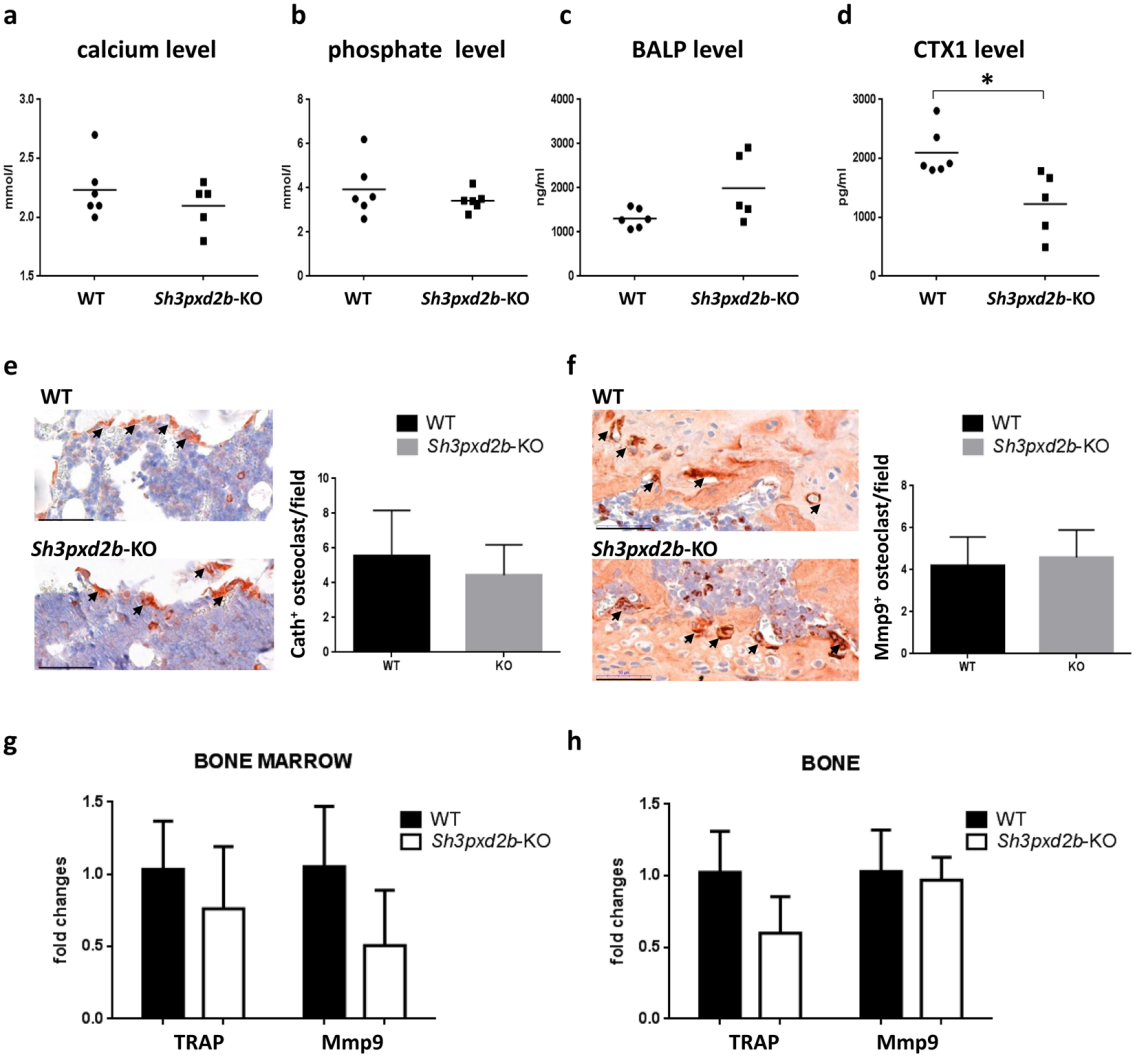
Examination of osteoclast markers. Measurements of the serum levels of calcium (**a**), phosphate (**b**), bone-specific alkaline-phosphatase (BALP) (**c**) and C-terminal telopeptide (CTX1) (**d**). The data were analyzed using Student’s t-test and 5–6 mice were used per genotype. The data are expressed as the mean ± SEM. (**e**) cathepsin-K-positive and (**f**) Mmp9-positive multinucleated osteoclast cells were quantified using IHC in the metaphyseal region of femurs from wild type and *Sh3pxd2b*-KO mice. Representative images of the stainings are shown. The number of osteoclasts/field in three wild type and three *Sh3pxd2b*-KO mice were analyzed, and the data are reported as the mean ± SEM. The scale bar represents 50 μm. The osteoclast marker RT-PCR measurements were performed separately in femur BM (**g**) and hard bone tissue (**h**). The expression levels of the TRAP and *Mmp9* bone resorption markers were measured. Three 7-month-old mice were used per genotype. The data are presented as the mean ± SEM, and the differences were analyzed by Student’s t-test. ^*^p < 0.05.

To further study the systemic effects of the observed osteoporotic phenotypes caused by the Tks4 mutation, we measured several biochemical serum parameters. The serum calcium and phosphate levels were within the normal ranges in the *Sh3pxd2b*-KO mice (similar to those of the wild type mice), indicating that the calcium balance was not severely disturbed and that the trabecular bone loss did not affect the systemic calcium and phosphate levels (Fig. 4a,b). Since osteoporosis-related pathologies are caused by an imbalance between the activities of osteoblasts and osteoclasts, the osteoporotic phenotype in the *Sh3pxd2b*-KO mice could be a consequence of impaired osteoclast or osteoblast functions. To address this possibility, we measured the serum levels of a bone formation marker, bone-specific alkaline phosphatase (BALP), and a bone resorption indicator, C-terminal telopeptide (CTX1)^21^. We did not detect a notable difference in the BALP serum levels (Fig. 4c). However, the CTX1 serum level was significantly lower in the *Sh3pxd2b*-KO mice compared with that of the wild type mice (Fig. 4d). To determine whether lack of Tks4 could affect the osteoclast population, we performed immunohistochemical analyses using osteoclast markers. Staining for cathepsin-K in distal femur sections showed no difference between the numbers of osteoclasts in the growth plate regions of the *Sh3pxd2b*-KO and wild type mice (Fig. 4e). The Mmp9^+^ multinucleated osteoclast cell populations were quantified, and the results showed that the populations of mature osteoclasts were similar in size in bones from *Sh3pxd2b*-KO and wild type mice (Fig. 4f). Furthermore, the *Mmp9* and TRAP expression levels (as measured by qPCR) were not significantly different in the bone tissue and bone marrow (BM) of the *Sh3pxd2b*-KO mice compared with their levels in the wild type mice (Fig. 4g,h). Although we showed that the CTX1 serum level was lower in the *Sh3pxd2b*-KO mice relative to that of the wild type mice, the osteoclast populations (based on the *Mmp9*, TRAP and cathepsin-K expression levels) were not significantly different between the *Sh3pxd2b*-KO and wild type mice. Thus, the osteoporotic phenotype of the *Sh3pxd2b*-KO mice was likely not due to increased osteoclast activity.

### Tks4 regulates osteoblastogenesis

**Figure 5:**
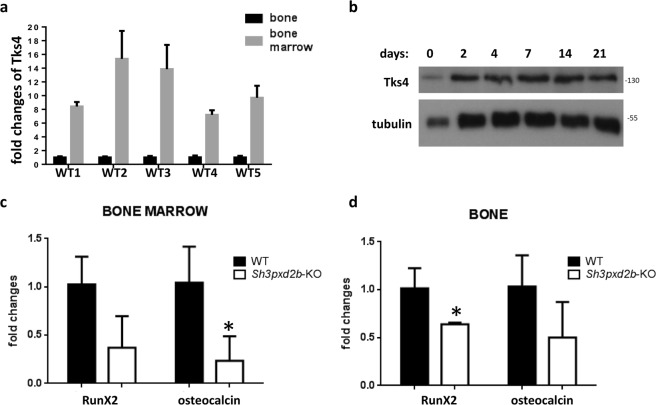
Examination of osteoblast markers. (**a**) The Tks4 expression levels were quantified separately in femoral hard bone tissue and BM by RT-PCR. The fold-change differences in the Tks4 mRNA levels were measured in five wild type mice (WT1-WT5), and the fold-changes were calculated using the 2^−ΔΔCt^ method. (**b**) BM-MSCs isolated from wild type mice were cultured and differentiated into the osteogenic lineage. Cell lysates were collected during the differentiation period on days 0, 2, 4, 7, 14, and 21, and the Tks4 protein levels were measured using western blotting. Tubulin was used as the loading control. The expression levels of the *RunX2* and osteocalcin bone formation markers were measured by RT-PCR separately in BM (**c**) and hard bone tissue (**d**). Three 7-month-old mice were used per genotype. The data are presented as the mean ± SEM, and the differences were analyzed by Student’s t-test. ^*^p < 0.05.

To measure Tks4 expression in adult bone, we first performed RT-PCR on bone and BM samples from wild type mice. Tks4 was expressed in both tissue types, although the relative Tks4 expression level was significantly higher in the BM fraction (Fig. 5a). Because we found higher Tks4 expression in the immature cell-type-enriched BM fraction compared to that in the terminally-differentiated hard bone tissue, we propose that loss of Tks4 might have a more pronounced effect in precursor cells than in mature cell types (such as osteocytes). Furthermore, we previously demonstrated that Tks4 is expressed in BM mesenchymal stem/stroma cells (BM-MSCs) and that MSCs derived from *Sh3pxd2b*-KO mice have a reduced capacity to differentiate into osteoblastic cells. Therefore, we measured the Tks4 expression level in osteogenic-differentiated wild type BM-MSCs to assess the presence of Tks4 during the osteoblast differentiation process. Western blotting was performed on cell lysates collected at six time points from *in vitro* osteogenic-differentiating BM-MSCs. The results showed that Tks4 protein was present at all of the tested time points during the BM-MSC differentiation process (Fig. 5b), revealing that the Tks4 is expressed throughout the BM-MSC osteoblastic differentiation program.

To learn more about the status of the osteoblasts in the bone and BM of the *Sh3pxd2b*-KO mice, we measured the expression levels of osteoblast-related marker genes^22,23^. As shown in Fig. 5c, the expression of *RunX2*, an early marker of osteoblast differentiation, was significantly down-regulated in the Tks4-deficient bone tissue compared to its level in wild type bone tissue. Moreover, the expression level of osteocalcin, a widely used marker for mature osteoblasts, was significantly lower in BM from the *Sh3pxd2b*-KO mice compared with that in BM from wild type mice (Fig. 5d). These findings are consistent with the previously suggested role of Tks4 in BM-MSC differentiation and support a model in which lack of Tks4 leads to an insufficient osteoblast supply. In addition to the normal osteoclast population in the bone of *Sh3pxd2b*-KO mice, we found that the potential of the BM-MSCs in the *Sh3pxd2b*-KO femurs to differentiate into the osteoblast lineage was impaired and that the expression levels of the osteoblast markers were reduced. One possible explanation for these phenotypes is that the cellular imbalance at the expense of bone-forming cells could lead to an osteoporotic phenotype of the *Sh3pxd2b*-KO mice.

## Discussion

Previous studies suggested that the Tks4 scaffold protein plays a role in bone biology. For example, mesenchymal stem cells with enhanced osteoblast differentiation potential have elevated Tks4 expression^13^. Furthermore, FTHS patients carrying mutations in *Sh3pxd2b* have skeletal dysplasia and craniofacial-bone dysmorphology^24^. A recent genome-wide mapping analysis of more than 700 outbred mice demonstrated that the gene encoding Tks4 (*Sh3pxd2b*) is a skull shape-determining quantitative trait locus (QTL), indicating that Tks4 is necessary for proper craniofacial bone shape development^25^. To elucidate the role of Tks4 in bone tissue formation and to investigate its functions in bone biology, we studied *Sh3pxd2b*-KO C57Bl/6 mice.

First, we compared detailed three-dimensional skull reconstructions reconstructed from micro-CT scans of wild type and *Sh3pxd2b*-KO mice. We found that the *Sh3pxd2b*-KO mice had domed craniums, brachycephalia, micrognathia, and wide fontanelle, features that are reminiscent of FTHS symptoms. There was a direct link between the skull morphologic alterations and the mutant genetic background in the Tks4-deficient mice, establishing this knock-out mouse strain as a valuable model system for studying craniofacial bone formation and maintenance.

Some mutations cause clinical symptoms related to craniofacial growth and result in abnormal osteogenesis in the long bones^26,27^. Although craniofacial dysmorphology is documented in Tks4-deficient FTHS patients, their skeletal bone phenotypes have not been examined in detail. Furthermore, an analysis of their bone microstructure is also lacking. The main aim of the current study was to examine the influence of Tks4 on skeletal phenotypes using micro-CT imaging. We show, for the first time, that loss of Tks4 leads to decreases in trabecular number and thickness that result in more porous bone tissue and osteoporotic bone trabecular structures in the femurs. Existing CBCT data from an FTHS patient were re-evaluated in this study, and the observations were consistent with the bone structure abnormalities in the *Sh3pxd2b*-KO mice. FTHS patients die during infancy or early childhood because of infections or heart failure. Consequently, it is difficult to obtain samples and examine the bone structure of adult FTHS patients, and it is impossible to conduct a large-scale examination of an FTHS cohort. Currently, only one adult FTHS patient has been documented^17^. CBCT was performed on his mandible at two time points due to dental problems. Reanalyzation of his CBCT data from a bone-structural perspective (i.e., cortical and cancellous bone) revealed abnormalities in the PMI and BV/TV and in the trabecular separation and total porosity parameters, which, taken together, indicate osteoporotic alterations. These results confirm that the bone abnormalities observed in the *Sh3pxd2b*-KO mice are highly reminiscent of the bone phenotypes of an adult FTHS patient, especially the alterations of the trabecular system.

The mechanisms by which Tks4 influences bone tissue remodeling remain obscure. We first examined the bone resorption component by measuring the levels of osteoclast-related markers in bone samples from *Sh3pxd2b*-KO mice. We did not detect any significant changes in the numbers of cathepsin-K- or *Mmp9*-stained osteoclasts. The expression levels of two osteoclast-related factors (TRAP, *Mmp9*) in the femurs of *Sh3pxd2b*-KO mice were also unaffected. Therefore, we concluded that the osteoporotic phenotype observed in the Tks4-deficient mice could not be caused by an expanded bone resorbing osteoclast cell population. Next, we measured the Tks4 expression levels in femurs and found higher Tks4 levels in the BM fraction compared with that of the hard bone tissue. This finding is particularly interesting given that Tks4 is required for the differentiation of BM-MSCs. In our previous study, we showed that BM-MSCs isolated from *Sh3pxd2b*-KO mice have impaired osteogenic differentiation capacity^13^. We also demonstrated that *Sh3pxd2b*-KO BM-MSCs had lower levels of the *RunX2* and Osterix master transcription factors and reduced deposition of calcium-containing minerals during *in vitro* osteogenic differentiation compared to these parameters in wild type cells. Moreover, our current results confirmed that Tks4 is present during *in vitro* differentiation of BM-MSCs into preosteoblasts and osteoblasts, reinforcing the notion that Tks4 influences osteoblast cell differentiation. We also showed that the expression levels of the early osteoblast differentiation marker *RunX2* and the late osteoblast-related marker osteocalcin were significantly reduced in the hard bone and BM of *Sh3pxd2b*-KO mice, respectively. Therefore, Tks4 may be connected to bone cell biology by functioning in BM-MSCs during osteoblast differentiation, perhaps regulating proper bone formation, rather than affecting bone resorption. This conclusion is consistent with the findings of previous studies linking osteoporosis to MSC differentiation defects^5,6,28–30^.

Based on the *in vitro* differentiation defect observed in the *Sh3pxd2b*-KO BM-MSCs and the changes in the *in vivo* expression levels of osteoblast-related transcription factors, we conclude that the differentiation of the MSC-derived osteoblastic cell lineage is disturbed in Tks4-deficient mice, leading to improper formation of the bone trabecular system.

## Methods

### Participants and cone beam CT (CBCT) measurements

We analyzed CBCT results of a male FTHS patient obtained when he was 21 and 24 years old. He was surgically treated to remove several impacted teeth and the CBCT data helped to follow his recovery. The description of the patient was previously published in a case report^17^. The bone microstructure analyses based on the CBCT data were performed by us and are published here for the first time. The control population consisted of 20- to 25-year-old healthy males. Informed consent was obtained from all participants before the CBCT examination. The subject exclusion criteria were evidence of osteoporosis, drug consumption, and anamnesis of any pathological disorder. We included CBCT data from ten participants in the control group. The measurements made in this study, including those involving human subjects, were conducted with ethical approval of the Regional and Institutional Committee of Science and Research Ethics, Semmelweis University, Budapest, Hungary (Ethics ID:138/2016). All methods involving measurements of humans were performed in accordance with the relevant guidelines and regulations of the Declaration of Helsinki for human research.

The CBCT measurements of the FTHS patient were performed at two time points (21 and 24 years old) in the Oral and Maxillofacial Radiology Department, Faculty of Dentistry at Istanbul University. We reanalyzed the CBCT data to characterize the mandibular bone tissue architecture. All of the measurements were pooled and included in the dataset (presented in Fig. 3). To analyze the cancellous bone component, six volumes of interest (VOIs) were chosen in the region between the apices of the canine and the premolars and the mandibular base, and these VOIs were analyzed using CTAn software (Bruker, Kontich, Belgium). Altogether, 40 different VOIs from the control group and 10 different VOIs from the FTHS patient were analyzed to calculate the bone volume/tissue volume ratio (BV/TV), the trabecular separation and the total porosity parameter statistics. The mandibular cortex measurement was performed according to Ledgerton’s statement^31^. The mental index (MI) and the distance from the lower border of the mandible to the lower border of the mental foramen (h) were measured on the left and right sides of the mandible. The MI/h ratios were calculated and are presented as the panoramic mandibular index (PMI). Altogether, 20 different PMIs from the control group and 4 different PMIs from the FTHS patient were measured and are presented in this study.

### Animals

The currently valid institutional and national guidelines for the care and use of animals were followed during this study. The animal procedures were conducted under the approval of the Pest County Government Office Directory of Food Chain Safety (Hungary) Ethics Committee (Approval number: PEI/001/2042-6/2014). The animals were maintained and handled in accordance with the Guidelines for Accommodation and Care of Animals (European Convention for the Protection of Vertebrate Animals Used for Experimental and Other Scientific Purposes). The *Sh3pxd2b*-KO mice (in the C57Bl/6 genetic background) were originally created by TaconicArtemis^13^. To generate these mice, the fifth and sixth coding exons of *Sh3pxd2b* gene were flanked by loxP sites. The floxed exons were then removed via Cre-mediated recombination in the germ line to inactivate *Sh3pxd2b*. *Sh3pxd2b*-KO mice are infertile; therefore, heterozygous animals were crossed to produce homozygous Tks4-deficient animals and wild type littermates for the experiments. The excision of the fifth and sixth exons was complete in all of the *Sh3pxd2b*-KO mice, and the genotype was confirmed in each generation using a previously described genomic PCR method^13^. Several tissue samples (skeletal muscle, heart muscle, brain, lung, spleen, adipose tissue) were tested by western blotting to confirm the absence of Tks4, and Tks4 was clearly absent in the *Sh3pxd2b*-KO mice^13^. Age-, strain- and sex-matched genotypically wild type and *Sh3pxd2b*-KO mice were used in all of the experiments.

### Cephalometric and micro-morphometric analyses using micro-computed tomography imaging

To quantify the craniofacial dimensions, skulls were dissected and removed from the mice and fixed in 10% formaldehyde. Formaldehyde-fixed femurs/tibias and skulls of age-matched wild-type and *Sh3pxd2b*-KO mice were imaged using a SkyScan 1172 (Bruker, SkyScan, Kontich, Belgium) micro-CT system. The scans were performed using a 5.03 μm isometric voxel size resolution under conditions of 50 kV and with a 200 (μA) X-ray source, using a 0.5-mm aluminum filter and a rotation step of 0.5°. Three-dimensional images were reconstructed and analyzed using the NRecon and CT-Analyser software. The length, width and height of each skull were measured. Landmark points were set according to a publication from the Richtsmeier laboratory^32^. The following landmark points were used: the cranium length was set between the basion (the midsagittal point on the anterior margin of the foramen magnum) and the intradentale superior (the most anterior midline point on the alveolar border superior to the septum between the central incisors); the cranium width was set between the right side and left side joining of the squamosal body to the zygomatic process of the squamosal; the cranium height was set between the basion (the midsagittal point on the anterior margin of the foramen magnum) and the intersection of the interparietal bones with the squamous portion of the occipital bone at the midline; the mandibular length was set between the tip of the mandibular angle and the inferior point on the mandibular symphysis. To accurately establish the landmark points on the cranium, we performed the measurements at three different time points and used the mean measurements for the statistical analysis. For the quantitative micro-morphometric measurements of the distal metaphysis of the femurs, 400 horizontal sections were analyzed starting 50 sections above the distal growth plate, and the boundaries of the trabecular area were selected^33^. The density threshold for the bone tissue was set manually by an experienced investigator. We measured the following parameters within each volume of interest (VOI): bone volume/tissue volume (BV/TV), trabecular number (TbN), trabecular thickness (TbTh), trabecular pattern factor (TbPf), and total porosity (TPo)^34^. The cortical bone parameters were evaluated in 100 slices toward the middle of the diaphysis, with the first slice taken 500 slices distal to the growth plate. A total of 6–6 mice were analyzed for each genotype in the 8-month-old group, and 3–3 mice were analyzed for each genotype in the 4-month-old group.

### Immunohistochemical analysis

The femurs and tibias were left connected to avoid damaging the knee joints, and the bones were cleaned of the muscles and fixed in paraformaldehyde. The bones were decalcified until they were soft and flexible then processed in paraffin. The embedded bones were sectioned longitudinally at a thickness of 2 μm. Serial sections were cut and stained with hematoxylin and eosin (H&E) or subjected to immunohistochemistry. The samples were deparaffinized in xylene and then hydrated in a series of aqueous alcohol solutions with decreasing alcohol concentrations. The sections were treated with H_2_O_2_ to block endogenous peroxidase activity, and antigen retrieval was performed by heat mediation. The slides were incubated in protein-blocking solution (Sniper blocking solution; Biocare Medical, Pacheco, CA) followed by incubation overnight at 4 °C with primary antibodies: rabbit polyclonal anti-Mmp9 (ab38898) or anti-cathepsin-K (ab19027) (Abcam). The sections were then incubated in Mach 2 Rabbit HRP Polymer (Biocare Medical) as the secondary antibody, and the colorimetric detection was performed with AEC (3-amino-9-ethylcarbazole). The immunostained sections were counterstained with hematoxylin. The negative control specimens from the experimental mice were bone sections treated in the same way but omitting the primary antibodies. No background staining was observed in any of the samples. The stained sections were digitally scanned with a Panoramic 250 Flash II high-resolution scanner (3DHISTECH Ltd., Budapest, Hungary). Osteoclasts were defined as multinucleated cells observed in bone lacunae in the metaphyseal region. The AEC-stained (Mmp9^+^ or cathepsin-K^+^) osteoclasts were quantified in four fields-of-view of a defined size per slide by annotating them using CaseViewer 2.1 software (3DHISTECH Ltd., Budapest, Hungary).

### Quantitative RT-PCR

Mice were euthanized and the femurs were removed. The muscle tissue was carefully cleaned from the isolated femurs, and the BM was flushed out with HBSS. The BM suspensions were centrifuged, and the BM cell pellets were kept at −80 °C until treatment with TRIzol™ Reagent (Life Technologies). The bones were snap-frozen and pulverized in liquid nitrogen and then homogenized in TRIzol™ Reagent. Total RNA was isolated separately from the bone and the BM samples using the Direct-zol® MiniPrep kit (Zymo Research) according to the manufacturer’s instructions. In-column DNase I treatment was used to prevent genomic DNA contamination. cDNA samples were reverse transcribed from 200 ng of RNA with the First Strand cDNA Synthesis kit for RT-PCR (Roche) using the provided random primers. The Pre-Developed GAPDH TaqMan® assay (Life Technologies) was used as the endogenous control. The TaqMan primers and probes (Thermo Fisher Scientific) used in this study were as follows: runt-related transcription factor 2 (*Runx2*) Mm00501584_m1, tartrate-resistant acid phosphatase TRAP *(Acp5*) Mm00475698_m1, bone gamma-carboxyglutamic acid-containing protein osteocalcin, (*Bglap*) Mm03413826_mH, matrix metalloproteinase 9 (*Mmp9*) Mm00442991_m1, glyceraldehyde-3-phosphate-dehydrogenase (*Gapdh*) Mm99999915_g1, Tks4 (*Sh3pxd2b*) Mm00616672_m1.

The RT-PCR analyses were carried out using a StepOne™ Real-Time PCR System (Life Technologies), and the mRNA fold-changes were calculated using the 2^−ΔΔCt^ method.

### Serum tests

Blood serum samples were collected from six-month-old wild type and *Sh3pxd2b*-KO mice in Microvette 500z-gel serum separator tubes (Sarstedt AG & Co., Nümbrecht, Germany). Following centrifugation, the clear serum was removed and stored at −80 °C. The serum concentrations of the bone biomarkers (calcium and phosphate) were analyzed using an Olympus AU480 (Beckman Coulter) chemistry system. The serum CTX1 and BALP levels were measured using commercially available ELISA assays (CTX1/CEA665Mu, Cloud-Clone Corp., Houston, TX, USA and BALP/CSB-E11914m, Cusabio Biotech, Wuhan, China) according to the manufacturers’ instructions.

### Osteogenic differentiation of BM-MSCs

Femurs and tibiae were removed from the mice and the BM cells were flushed out. The washed BM cell suspensions were seeded in culture flasks (BD Falcon, Bedford, MA) in DMEM F12 medium (Thermo Fisher Scientific, Bremen, Germany) supplemented with 10% FBS and 5% horse serum (Invitrogen). After two days, the non-adherent cells were removed, and the adherent cells were further propagated for characterization of the BM-MSC phenotypes (as previously described and demonstrated in^13^). Osteogenic differentiation was induced using DMEM supplemented with 10% FCS, 10 mM β-glycerophosphate (Sigma-Aldrich), 50 μg/ml ascorbic acid (Sigma) and 0.01 μM hydrocortisone (Sigma-Aldrich). The cultures were incubated for 21 days and the medium was changed every 3 days.

### Western blot analysis

Differentiated BM-MSC cells were collected for western blotting during osteogenic differentiation on days 0, 2, 4, 7, 14, and 21. The cells were lysed and the lysates were clarified by centrifugation. Equal volumes of the samples were subjected to SDS-PAGE, and the proteins were transferred to PVDF membranes. The membranes were blocked and incubated overnight with anti-Tks4 (polyclonal rabbit) or anti-alpha-tubulin (monoclonal mouse, Sigma-Aldrich, USA) primary antibodies. Polyclonal anti-Tks4 specific antibody was generated earlier^11^. After several washes, the membranes were incubated with HRP-conjugated secondary antibodies (GE Healthcare, Lottle Chalfont, Buckinghamshire, UK). The reactive antigens were visualized with ECL detection reagents (Amersham Life Science, Buckinghamshire, UK).

### Statistical analysis

All of the results are presented as mean ± SEM. Differences between two experimental groups (e.g., wild type vs. *Sh3pxd2b*-KO) were compared using unpaired Student’s t-tests. Graphpad Prism version 5.0 software was used for the statistical analyses, and differences were considered significantly different at p < 0.05.
